# A Comparative Study of Machine Learning Methods for Predicting Live Weight of Duroc, Landrace, and Yorkshire Pigs

**DOI:** 10.3390/ani12091152

**Published:** 2022-04-29

**Authors:** Alexey Ruchay, Svetlana Gritsenko, Evgenia Ermolova, Alexander Bochkarev, Sergey Ermolov, Hao Guo, Andrea Pezzuolo

**Affiliations:** 1 Federal Research Centre of Biological Systems and Agro-Technologies of the Russian Academy of Sciences, 460000 Orenburg, Russia; ran@csu.ru; 2 Department of Mathematics, Chelyabinsk State University, 454001 Chelyabinsk, Russia; 3 Agricultural Product Production and Processing Technology Department, South Ural State Agrarian University, 457100 Troitsk, Russia; zf.usavm@mail.ru (S.G.); zhe1748@mail.ru (E.E.); a.k.bochkarev@mail.ru (A.B.); sergey.ermolov@bk.ru (S.E.); 4 College of Land Science and Technology, China Agricultural University, Beijing 100083, China; guohaolys@cau.edu.cn; 5 Department of Land, Environment, Agriculture and Forestry, University of Padova, 35020 Legnaro, Italy

**Keywords:** live pig weight estimation, machine learning, ensemble methods, prediction, regression algorithm, body measurement

## Abstract

**Simple Summary:**

Live weight is an important indicator of livestock productivity and serves as an informative measure for the health, feeding, breeding, and selection of livestock. In this paper, the live weight of pig was estimated using six morphometric measurements, breed, weight at birth, weight at weaning, and age at weaning. In the present paper, we propose a comparative analysis of various machine learning methods using outlier detection, normalisation, hyperparameter optimisation, and stack generalisation to increase the accuracy of the predictions of the live weight of pigs. The StackingRegressor algorithm yielded a prediction quality of the live weight of Duroc, Landrace, and Yorkshire pigs that was higher than that of the state-of-the art algorithms.

**Abstract:**

Live weight is an important indicator of livestock productivity and serves as an informative measure for the health, feeding, breeding, and selection of livestock. In this paper, the live weight of pig was estimated using six morphometric measurements, weight at birth, weight at weaning, and age at weaning. This study utilised a dataset including 340 pigs of the Duroc, Landrace, and Yorkshire breeds. In the present paper, we propose a comparative analysis of various machine learning methods using outlier detection, normalisation, hyperparameter optimisation, and stack generalisation to increase the accuracy of the predictions of the live weight of pigs. The performance of live weight prediction was assessed based on the evaluation criteria: the coefficient of determination, the root-mean-squared error, the mean absolute error, and the mean absolute percentage error. The performance measures in our experiments were also validated through 10-fold cross-validation to provide a robust model for predicting the pig live weight. The StackingRegressor model was found to provide the best results with an MAE of 4.331 and a MAPE of 4.296 on the test dataset.

## 1. Introduction

Live weight is an important indicator of livestock productivity and serves as an informative feature for the health, feeding, breeding [1], and selection of livestock [2]. The prediction of live animal weight based on different body characteristics observed during different growth periods for sheep [3], goats [4], chickens [5], ducks [6], rams [7], and cattle [8] has been extensively studied in the literature. Moreover, live weight measurement is a production tools available to farmers in nutrition [9], fertility management [10], health [11], and marketing [12]. Live weight prediction can be based on morphological features that are automatically measured using computed tomography [13], ultrasonic machines [14], and 3D vision systems [15].

Although the direct weighing method provides the most accurate [16] results, it requires physical effort to force the animal to stand on the scale. Therefore, this method is traumatic and can be stressful for both animals and livestock keepers. The latter is also true for other contact methods of weight measurement, such as measuring the pig’s girth behind the front legs. Current digital noncontact weight measurement methods are limited by the requirement that the animal be in some standard position and remain motionless during capture with a 2D CCD camera [16,17,18] or a 3D camera [19]. That is, such requirements are feasible in a research laboratory, but not on an active pig farm. Animal weight estimates are computed from measurements of the animal’s back area and height, which are estimated using 2D digital images from a CCD camera or animal volume estimates from a 3D camera. The error rate of animal weight estimation by these laboratory methods is approximately 5–7%. The three-dimensional structure of the animal makes it possible to select the best animals for breeding, culling, and quality control of various cuts of meat from live pigs. For example, leg weakness is a major cause of premature culling of breeding sows, which can be automatically determined by the shape and position of the legs [20]. Note that inspectors’ visual assessments may vary due to fatigue and a lack of training. Many properties of the three-dimensional structure can be extracted from two-dimensional images, while three-dimensional data allow for the isolation of cross-sectional areas and volumes and measurement of characteristics such as the rectangularity of the back muscles, which are known measures of muscle mass. With regular measurement of the three-dimensional shape of an animal, it is possible to routinely quantify the effects of an animal’s height, diet, genetics, health, and posture. There are two ways to obtain the three-dimensional shape of an animal: using stereo [20] or depth cameras [21,22]. However, restrictions on the position of the animal and its immobility remain, and therefore, the application of such systems on active pig farms is not possible.

In past studies, linear regression analysis was usually used to predict the live weight of pigs [23,24,25]; however, these traditional methods are inadequate for prediction [3]. Recently, several researchers have effectively employed various machine learning algorithms to predict the live weight of pigs using morphological features [26]. These methods aim to predict the live weight of animals from morphological measurements. These studies have shown the potential of machine learning algorithms for accurately predicting the nonlinear relationship between the body weight and morphological traits of animals [3].

The objective of this paper is to study various machine learning methods [27,28] for predicting the live weight of pigs based on breed, weight at birth, weight at weaning, age at weaning, and six morphometric measurements. This study aimed to identify the best machine learning algorithms for predicting the live weight of pigs using various morphological features.

The main contributions of this article are as follows:

- We show that machine learning methods can provide better results than traditional linear regression algorithms for predicting the live weight of pigs.
- By using outlier detection, normalisation, hyperparameter optimisation, stack generalisation, and cross-validation, pig live weight prediction was improved.
- The dataset and model for live weight prediction of Duroc, Landrace, and Yorkshire pigs can be downloaded for use by the livestock research community freely by following the link [29].

## 2. Materials and Methods

The animal experimental and data collection were approved by the Animal Care and Use Committee of the South Ural State Agrarian University and Federal Research Centre of Biological Systems and Agro-technologies of the Russian Academy of Sciences (01-14/758). All procedures and data collection in this study were conducted according to the Guidelines for Experimental Animals (Russia).

### 2.1. Data Collection

**Figure 1:**
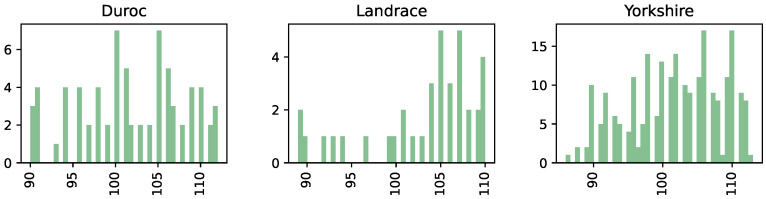
Histogram of the distribution of the live weight by breed.

This study used data from 340 Duroc, Landrace, and Yorkshire pigs kept on a private farm in the Chelyabinsk region of Russia. Traits associated with reduced live weight of pigs included weight at birth (kg), weight at weaning (kg), age at weaning (days), body length (cm), chest girth (cm), withers height (cm), chest depth (cm), chest width (cm), and metacarpus girth (cm). The weight of these pigs was measured with a scale and ranged from 86 to 113 kg. The distribution of pigs by breed was as follows: 231 Yorkshire, 72 Duroc, 37 Landrace. The age of the pigs was 6 months. They were all females at finishing stages. A histogram illustrating the distribution of the live weight by breed is shown in Figure 1.

**Figure 2:**
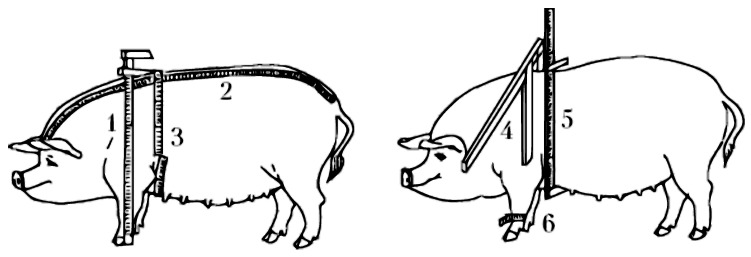
Picture of six measured body dimensions of a pig: (1) body length, (2) chest girth, (3) withers height, (4) chest depth, (5) chest width, and (6) metacarpus girth.

The six body measurements shown in Figure 2 were taken manually by an expert with tailor measuring tape and measuring sticks and recorded in centimetres. The created data collection are open and available to the research community [29].

The estimated body measurements were as follows:

- Body length was measured at the middle of the occipital ridge along the upper straight line of the neck, withers, back, loin, and sacrum to the root of the tail using tailor tape.
- Chest girth was measured behind the shoulder blades by girdling the animal in a vertical plane tangent to the posterior angles of the shoulder blades using tailor tape.
- Withers height was measured at the highest point of the withers using a measuring stick.
- Chest depth was measured from the withers to the sternum vertically, tangent to the posterior angle of the scapula, using a measuring stick.
- Chest width was measured at the widest point of the vertical tangent to the posterior angle of the scapula using a measuring stick.
- Metacarpus girth was measured at the lower end of the upper third of the metacarpus using tailor tape.

### 2.2. Preprocessing

Outlier detection as a preprocessing step was used to identify anomalies of rare samples that were suspicious because they differed significantly from most data points. The SciKit-Learn library (SKlearn) [30] provides a set of machine learning tools to detect outliers: *z*-score, InterQuartileRange, IsolationForest, LocalOutlierFactor, OneClassSVM, EllipticEnvelope. After extensive experimentation with normalisation algorithms, the *z*-score normalisation algorithm was chosen because it exhibited the best performance. The *z*-score normalisation algorithm calculates the *z*-score for each sample of data. The *z*-score is defined as

(1) $$z=\frac{x-u}{s},$$

where *x* is the current sample value and *u* and *s* are the mean and standard deviation of all samples, respectively.

### 2.3. Feature Standardisation

The features have different units and scales. To reduce this impact on the prediction results, the data should be normalised before training the model to make sure that each feature has the same order of magnitude.

The following normalisation algorithms from the SKlearn library [30] were used: MinMaxScaler, MaxAbsScaler, StandardScaler, PowerTransformer, StandardScaler, PowerTransformer, QuantileTransformer, Normalizer, FunctionTransformer, PolynomialFeatures, and RobustScaler. The MinMaxScaler normalisation algorithm was chosen because it exhibited the best performance. The StandardScaler normalisation algorithm scales each feature to a specified range. The normalisation is shown as:

(2) $$y=\frac{2(x-x_{min})}{x_{max}-x_{min}}-1,$$

where *x* represents the sample value, $x_{min}$ and $x_{max}$ represent the minimum and maximum values of all samples, respectively, and *y* is the normalised value of the feature.

### 2.4. Machine Learning Algorithms

We studied all the machine learning algorithms from the SKlearn library [30]. However, some algorithms led to poor results, which are not shown in this article. The model evaluation results are presented only for the following regression algorithms: extra trees (ExtraTreesRegressor), random forest (RandomForestRegressor), k-nearest neighbours (KNeighborsRegressor), linear regression (LinearRegression), epsilon-support vector (SVR), gradient boosting (GradientBoostingRegressor), decision tree (DecisionTreeRegressor), adaptive boosting (AdaBoostRegressor), ridge regression with cross-validation (RidgeCV), cross-validated lasso linear model (LassoCV), cross-validated lasso with the LARS algorithm (LassoLarsCV), cross-validated orthogonal matching pursuit model (OrthogonalMatchingPursuitCV), Bayesian ridge (BayesianRidge), Theil–Sen estimator (TheilSenRegressor), and linear regression Huber model (HuberRegressor). The following machine learning algorithms were also used for prediction: two methods of gradient boosting (CatBoostRegressor) [31] and (LGBMRegressor) [32], as well as scaled gradient boosting (XGBRegressor) [33].

The data were initially partitioned randomly into two parts: the training dataset (70%) and the test dataset (30%). Additionally, 20% of the training dataset were used for validation.

Our experiments with the models involved testing various combinations of hyperparameters to find the optimal response. We used the GridSearchCV algorithm from the SKlearn library [30] to automate the process of obtaining the best combination of hyperparameters. We found the optimal hyperparameters for all regression algorithms used using GridSearchCV. Some algorithms can lead to overfitting, especially tree-based methods. Therefore, we used regularisation and the early stopping technique to avoid overfitting.

### 2.5. Ensemble Methods

Ensemble methods have greatly helped obtain a more powerful prediction based on combinations of many different machine learning models. There are various ensemble methods: averaging methods based on different weighted averaging algorithms, bagging, boosting, stack generalisation, and the special network StackNet. StackNet is a scalable meta-modelling methodology that utilises stacking to combine multiple models in a neural network architecture with multiple levels in parallel.

Stacked generalisation or stacking is an ensemble machine learning algorithm [34]. The advantage of stacked generalisation is the capability of prediction with better performance than any single model. Stack generalisation typically yields better performance than any single trained model [35]. Although stack generalisation does not guarantee an improvement in performance, it depends on the complexity of the regression task, the choice of base models, and uncorrelated base models in predictions.

Stacked generalisation uses a meta-learning algorithm to fit a combination of the prediction models. Stacked generalisation can combine the predictions from some models on the same dataset, such as boosting and bagging. In contrast to boosting, the stacking model uses a single model to fit a combination of the predictions from the models. In contrast to bagging, the stacking model is typically different and fits the same dataset. The stacking model consists of some base models and a meta-model that integrates the predictions of the base models. The outputs from the base models are used as the input to the meta-model. In the meta-model, the training dataset can be prepared using k-fold cross-validation from the base models and can also use the training dataset as the inputs to the base models, which can provide additional data to the meta-model to fit the best combination of the predictions from the meta-model. The base models are trained on the entire original training dataset, and the meta-model is trained on the prepared training dataset. Base models should be diverse and complex.

### 2.6. Model Evaluation

Some evaluation criteria were used to estimate the performance of the models used in this study for predicting the pig live weight.

In this study, we examined various commonly used evaluation measures. We used the coefficient of determination ($R^{2}$), the root-mean-squared error (RMSE), the mean absolute error (MAE), and the mean absolute percentage error (MAPE) as measures to evaluate quality. They are defined as

(3) $$R^{2}=1-\frac{\sum_{i=1}^{n}{\left(y_{i}-f_{i}\right)}^{2}}{\sum_{i=1}^{n}{\left(y_{i}-\bar{y}\right)}^{2}},$$

(4) $$\mathrm{RMSE}=\sqrt{\frac{1}{n}\sum_{i=1}^{n}{\left(y_{i}-f_{i}\right)}^{2}},$$

(5) $$\mathrm{MAE}=\frac{1}{n}\sum_{i=1}^{n}\left|y_{i}-f_{i}\right|,$$

(6) $$\mathrm{MAPE}=\frac{1}{n}\sum_{i=1}^{n}\left|\frac{y_{i}-f_{i}}{y_{i}}\right|,$$

where *n* is the number of samples in the dataset, $\bar{y}$ presents the average value among the measured live weight values, $y_{i}$, i=1,…,n are the measured live weight values, and $f_{i}$, i=1,…,n are the predicted live weight values.

## 3. Results and Discussion

### 3.1. Preprocessing

**Figure 3:**
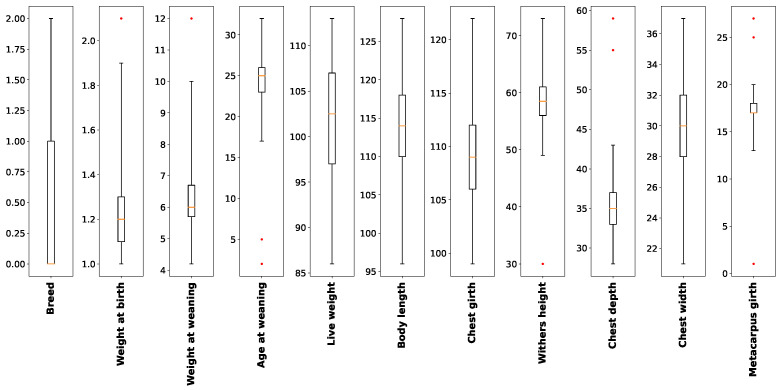
In the boxplots, the whiskers show the range, the boxes show the upper and lower quartile and median (solid dark horizontal line) values, and red points are outliers.

After preprocessing, 311 out of 340 samples remained after excluding outliers. Figure 3 shows the boxplots before preprocessing. Note that body weight, age at weaning, live weight, body length, withers height, chest depth, and metacarpus girth have outliers because of anomalous causes, for example an error in data transcription, human error, or natural deviations in populations. The basic statistics of the features used after preprocessing are shown in Table 1.

### 3.2. Machine Learning Model

We obtained the results of various evaluation measures used to evaluate model performance on the training and test datasets shown in Table 2. The tree-based algorithms improved the overfitting situation. The most robust algorithm was actually the LassoCV algorithm with a consistent $R^{2}$ of approximately 0.299 on the training dataset and 0.301 on the test dataset, but the RidgeCV algorithm did not have the smallest MAE of 4.533 and MAPE of 4.521 on the test dataset.

We investigated different ensemble methods to achieve better predictions based on combinations of many different machine learning models (Show in Table 3): the averaging method using VotingRegressor method [30], bagging using the BaggingRegressor method [30], and stack generalisation using the StackingRegressor method [30]. Our experiments with models involved testing various combinations of hyperparameters and machine learning algorithms to find the optimal response using an exhaustive search. We used a varied range of models: RandomForestRegressor, ExtraTreesRegressor, DecisionTreeRegressor, AdaBoostRegressor, XGBRegressor, CatBoostRegressor, KNeighborsRegressor, LassoCV, and RidgeCV.

The RidgeCV base model with two estimators yielded the optimal response for the BaggingRegressor model. The KNeighborsRegressor, LassoCV, and RidgeCV base models yielded the optimal response for the VotingRegressor model. The StackingRegressor model yielded the optimal response using LassoCV, KNeighborsRegressor, and LGBMRegressor base models and the CatBoostRegressor meta-model. We obtained the following results shown in Table 2 for the ensemble models. The most robust algorithm was the StackingRegressor algorithm, with a consistent $R^{2}$ of approximately 0.377 on the training dataset and 0.352 on the test dataset. Moreover, the StackingRegressor algorithm had the smallest MAE of 4.331 and MAPE of 4.296 on the test dataset.

The performance measures in our experiments were also validated by 10-fold cross-validation. The 10-fold cross-validation results for the StackingRegressor model using various evaluation measures are shown in Table 4. For all 10 iterations, the values of the evaluation measures remained almost the same, indicating the stability of the StackingRegressor model for prediction. Thus, we can conclude that the StackingRegressor model performed better than the other models used in this study to predict pig live weight.

**Figure 4:**
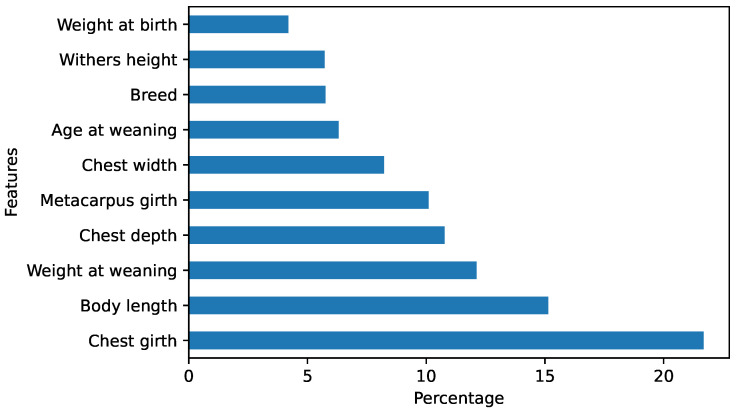
Feature importance identified by the StackingRegressor algorithm.

Figure 4 shows the feature importance identified by the StackingRegressor algorithm for predicting the pig live weight. The most important feature was found to be chest girth, which accounted for approximately 21% of the variation in the pig live weight prediction. Body length and weight at weaning were also found to be important features, together explaining approximately 27% of the variation. Other features such as metacarpus girth and chest depth contributed little to the variation.

It is of interest to note that the StackingRegressor algorithm yielded a prediction quality of the live weight of Duroc, Landrace, and Yorkshire pigs that was higher than that of the state-of-the art algorithms [3,23,24,25,26].

## 4. Conclusions

This study employed various machine learning algorithms to predict the live weight of Duroc, Landrace, and Yorkshire pigs using body length, chest girth, withers height, chest depth, chest width, metacarpus girth, weight at birth, weight at weaning, and age at weaning. We found strong evidence of better performance for machine learning algorithms compared with the traditional linear model using various evaluation measures. The StackingRegressor model was found to provide more accurate pig live weight prediction, outperforming the traditional linear model. The results of the present study demonstrate that the StackingRegressor model can be used to predict pig live weight. Moreover, outlier detection, normalisation, hyperparameter optimisation, and stack generalisation algorithms can be used to increase the accuracy of predicting pig live weight. The findings of this study may help researchers and practitioners adopt machine learning algorithms for accurate live weight prediction using various morphological traits and other features. Since we used data from pigs with the weight ranging from 86 to 113 kg, the proposed model will be guaranteed to predict the weight of pigs in this range. We think that an indirect automated estimation of the live weight should be a non-invasive measurement of morphometric measurements based on computer vision, followed by live weight prediction using a machine leaning.

## Figures and Tables

**Table 1:** The mean values, standard deviation (SD), and coefficient of variation (CV) of each feature.

Features	Mean	SD	CV (%)
Live weight (kg)	101.78	6.51	6.40
Weight at birth (kg)	1.21	0.12	10.13
Weight at weaning (kg)	6.11	0.77	12.61
Age at weaning (days)	24.56	3.04	12.37
Body length (cm)	113.73	5.27	4.63
Chest girth (cm)	109.27	4.35	3.98
Withers height (cm)	58.78	3.30	5.61
Chest depth (cm)	35.12	2.88	8.19
Chest width (cm)	30.11	2.58	8.56
Metacarpus girth (cm)	17.37	0.82	4.74

**Table 2:** Comparison of the ensemble model performances in terms of $R^{2}$, RMSE, MAE, and MAPE.

Algorithm	On Training Dataset				On Testing Dataset			
	$R^{2}$	RMSE	MAE	MAPE	$R^{2}$	RMSE	MAE	MAPE
VotingRegressor	0.394	5.026	4.172	4.150	0.328	5.436	4.594	4.573
BaggingRegressor	0.300	5.403	4.432	4.399	0.303	5.539	4.504	4.487
StackingRegressor	0.377	5.095	3.803	3.803	0.352	5.339	4.331	4.296

**Table 3:** Comparison of the model performances in terms of $R^{2}$, RMSE, MAE, and MAPE.

Algorithm	On Training Dataset				On Testing Dataset			
	$R^{2}$	RMSE	MAE	MAPE	$R^{2}$	RMSE	MAE	MAPE
RandomForestRegressor	0.652	3.811	3.125	3.101	0.264	5.688	4.798	4.777
ExtraTreesRegressor	0.588	4.145	3.389	3.362	0.247	5.755	4.903	4.881
KNeighborsRegressor	0.443	4.817	3.851	3.828	0.232	5.812	4.884	4.858
LinearRegression	0.313	5.354	4.431	4.405	0.282	5.619	4.607	4.592
GradientBoostingRegressor	0.756	3.192	2.572	2.551	0.260	5.706	4.757	4.701
AdaBoostRegressor	0.571	4.229	3.725	3.674	0.224	5.842	4.865	4.823
RidgeCV	0.307	5.374	4.437	4.410	0.297	5.561	4.533	4.521
LassoCV	0.299	5.408	4.465	4.438	0.301	5.545	4.542	4.532
LassoLarsCV	0.271	5.514	4.609	4.585	0.269	5.670	4.704	4.698
BayesianRidge	0.272	5.508	4.530	4.504	0.305	5.528	4.577	4.566
TheilSenRegressor	0.275	5.498	4.481	4.467	0.208	5.901	4.822	4.808
XGBRegressor	0.714	3.454	2.820	2.768	0.248	5.751	4.748	4.675
LGBMRegressor	0.801	2.877	2.239	2.222	0.270	5.667	4.720	4.667
CatBoostRegressor	0.786	2.986	2.422	2.408	0.288	5.596	4.692	4.658

**Table 4:** Results of 10-fold cross-validation for the most efficient algorithms on the test dataset. SD ($\times 10^{-4}$) is the standard deviation.

Algorithm	$R^{2}$		RMSE		MAE		MAPE	
	Mean	SD	Mean	SD	Mean	SD	Mean	SD
StackingRegressor	0.369	0.027	5.226	0.037	4.319	0.028	4.281	0.019

## Data Availability

The data presented in this study are available.
